# The efficacy, feasibility, and technical outcomes of a GPT-4o-based chatbot Amanda for relationship support: A randomized controlled trial

**DOI:** 10.1371/journal.pmen.0000411

**Published:** 2025-09-24

**Authors:** Laura M. Vowels, Matthew J. Vowels, Shannon K. Sweeney, S. Gabe Hatch, Joëlle Darwiche

**Affiliations:** 1 Institute of Psychology, University of Lausanne, Lausanne, Switzerland; 2 School of Psychology, University of Roehampton, London, United Kingdom; 3 The Sense Innovation and Research Center, Lausanne and Sion, Switzerland; 4 Radiology Department, Lausanne University Hospital and University of Lausanne, Lausanne, Switzerland; 5 Centre for Vision Speech and Signal Processing, University of Surrey, Guildford, United Kigndom; 6 Hatch Data and Mental Health, Payson, Utah, United States of America; 7 FAmily and DevelOpment research center (FADO), Institute of Psychology, University of Lausanne, Lausanne, Switzerland; National University of Singapore, SINGAPORE

## Abstract

This randomized controlled trial evaluated the efficacy, feasibility, and technical outcomes of Amanda, a GPT-4-based chatbot, in delivering single-session relationship interventions. A total of 258 participants were randomly assigned to engage with either Amanda (*n* = 130) or a writing task (*n* = 128) focused on conflict reappraisal. Findings demonstrated significant improvements across 13 of 14 outcome variables—including relationship satisfaction, communication patterns, dyadic coping, problem-specific confidence, and individual well-being—over time in both conditions. Improvements emerged immediately after the intervention and were sustained or continued to improve at the two-week follow-up. However, there were no significant group differences for most outcomes, suggesting that both interventions were comparably effective. One significant group-by-time interaction emerged: participants in the chatbot condition reported lower levels of the partner-demand/self-withdraw communication pattern immediately post-intervention. The writing condition was also associated with lower overall distress about the issue. Feasibility outcomes indicated strong participant engagement with Amanda. Usability was rated highly (M = 4.19/5), as were therapeutic skills (M = 3.99/5) and working alliance (M = 4.75/6). Technical evaluation of interaction transcripts supported these findings, with high coder agreement on Amanda’s empathy, therapeutic questioning, and coherence. However, limitations were noted: Amanda occasionally produced repetitive or generic responses and did not consistently identify potential safety concerns. Overall, results suggest that Amanda provides a feasible and effective single-session relationship intervention, comparable in impact to an evidence-based writing task. This study highlights the potential for large language model-based chatbots to deliver scalable, accessible relationship support. Future research should assess Amanda’s use in multi-session interventions, explore performance in clinical populations, and enhance risk detection capabilities to ensure safe deployment in real-world settings.

## Introduction

Relationship distress is a prevalent issue, with approximately one in three couples experiencing clinically significant problems that impact multiple aspects of life [1–4]. Traditional couple therapy is well-established as an effective intervention [5,6]. However, numerous barriers, including financial constraints and societal stigma, prevent many couples from accessing these services [3,7]. In response to these challenges, online interventions have emerged as a promising alternative, offering a more accessible and flexible means of support [7–10]. Research has shown that online interventions can be as effective as face-to-face interventions [10,11]. Despite their potential, digital interventions face significant challenges, most notably high dropout rates, which call into question their long-term feasibility [12,13].

Recent advancements in generative artificial intelligence (GenAI) and large language models (LLMs) have opened new avenues for developing interventions, including providing relationship support. GenAI and LLMs, which encompass machine learning algorithms that can generate novel human-like text outputs, form the backbone of modern chatbots. Chatbots have demonstrated potential in mental health interventions [14–20], fostering therapeutic alliance that support sustained engagement [18,21]. For instance, a content analysis of the Wysa chatbot revealed that users developed strong bonds with the bot, with therapeutic alliance scores comparable to those in human-therapist interactions [22]. A systematic review emphasized that personalization and empathetic responses are critical for improved mental health outcomes in chatbot interactions [16]. Nonetheless, misinformation in sensitive contexts like mental health treatment could lead to harmful outcomes. Therefore, research must ensure the consistency and reliability of these chatbots.

The release of OpenAI’s ChatGPT, particularly the GPT-4 model, represents a substantial advancement in this field, potentially overcoming these earlier challenges. GPT-4, which builds on the foundation laid by GPT-3.5, offers more nuanced and human-like conversational capabilities, making it a strong candidate for developing more effective and engaging online interventions. GPT-4 has demonstrated improved performance over previous models in medical examinations and genetic information delivery [23,24]. However, challenges persist, including the generation of inaccurate information and outdated content [23,25]. In mental health and substance use education, GPT-4’s outputs required human oversight and were substandard compared to expert-developed materials [25]. Across other studies, research has shown that LLMs like GPT-3.5 and GPT-4 may outperform physicians and psychotherapists in providing information which is perceived as empathic and helpful [26,27]. In a recent study with a small sample, seven psychiatric inpatients interacted in 3–6 sessions provided by ChatGPT: The participants reported higher quality of life compared to the control group (six patients) and reported high satisfaction with the chatbot [28]. To our knowledge no other studies have directly examined the efficacy of modern LLMs in providing mental health interventions, nor have they examined how LLMs perform relative to other previously established relationship interventions. Thus, while LLMs like GPT-4 show potential in various interventions, including improving user engagement, their use requires careful consideration of limitations and ethical implications to ensure patient safety and information accuracy [25].

Initial studies evaluating the potential of GPT-4 for relationship interventions suggest that it may offer a viable alternative to traditional therapy. For example, responses generated by GPT-4 were rated as more empathetic and helpful than those produced by relationship experts in simulated sessions [27]. In a single session relationship intervention using GPT-4, participants and researchers rated the chatbot high across different therapeutic skills with 85% of the 20 participants reporting having had a positive experience with the chatbot [29]. This reflects a broader trend where LLMs are increasingly being explored for their therapeutic potential across various fields, although their application to relationship therapy is still in its early stages.

Given the rapid advancement of AI technologies and the increasing interest in their application to therapeutic settings, it is crucial to systematically investigate the capabilities and limitations of LLM-powered chatbots in providing relationship interventions and compare their efficacy to previously established relationship interventions. This study aims to address this gap by exploring how GPT-4 can deliver single-session relationship interventions, how it compares to an established writing intervention of similar length, and examining users’ perceptions of these chatbot interactions. While LLM-powered chatbots have shown promise, there remains a need for more sophisticated models specifically tailored for relationship support. Amanda, a GPT-4o-based chatbot, is designed to fill this gap by integrating a specialized prompt that instructs the AI to act as a relationship therapist, reflecting on client statements, providing empathy, and asking pertinent follow-up questions [29]. She has been evaluated across a range of therapeutic skills, including empathy, realism, and collaborative approach, based on frameworks established in prior studies [14,29] and has been shown to provide a feasible solution for relationship support [29].

There are three main types of outcomes that can be used to evaluate chatbot performance: Efficacy, feasibility, and technical outcomes [18]. A recent systematic review showed that while 61% of studies on chatbots reported clinical outcomes and 37% report feasibility outcomes, only 1% reported technical outcomes [18]. This is a significant gap in the literature as evaluating technical outcomes is imperative to establish the performance and safe usage of chatbots for clinical interventions. The present study employs a robust methodology evaluating all three types of outcomes.

First, we included a set of validated questionnaires for establishing efficacy of the intervention including a wide range of clinical outcomes such as relationship satisfaction, communication skills, dyadic coping, and individual well-being. We also included problem specific outcomes such as hopefulness, distress, and confidence about the presenting problem. The clinical outcomes were compared across the chatbot, and an established writing condition focused on conflict reappraisal to provide a comparison between a more traditional self-help intervention and a more interactive chatbot interaction [30]. Second, we also evaluated the feasibility of the chatbot intervention, i.e., the usability of the chatbot including how users view the interactions with the chatbot, using previously validated questionnaires on chatbot usability and therapeutic alliance as well as specific therapeutic skills using a questionnaire from previous work on relationship support chatbots [27,29]. Finally, to assess technical outcomes, i.e., outcomes related to the performance of the chatbot such as coded empathy, human-likeness, and conversation depth, we coded the transcripts of the interactions between the participants and the chatbot for technical outcomes using an evaluation framework based on prior work [14,29]. This evaluation framework helps to address the technical challenges that have historically limited the effectiveness of AI in therapeutic roles. By systematically assessing Amanda’s performance across these metrics using a variety of methodologies, this study seeks to advance our understanding of how AI can be effectively integrated into relationship interventions, potentially transforming how couples access and benefit from therapeutic support.

In this preregistered trial, participants were randomly assigned to engage with Amanda or complete a brief social psychological writing task focused on conflict reappraisal shown to improve relationship outcomes [30]. We preregistered the following two main hypotheses:

H1: Participants in both treatment arms (chatbot and writing task) will report significant improvements over time (from pre-intervention to post-intervention and/or follow-up) in a) relationship satisfaction, b) relationship confidence, c) individual well-being, d) hopefulness about the issue, e) confidence in resolving the issue, f) communication about the issue, and g) distress related to the issue. These within-subject improvements are expected as both interventions are designed to promote cognitive and emotional reflection on a specific relationship problem. Given both conditions help the participants address and think differently about their relationship issue, we expect them to feel better about their relationship directly after the intervention. However, we might expect that there is a greater improvement at two-week follow-up given the participants will have had time to engage with their partner following the intervention.
H2: The chatbot intervention (Amanda) will lead to significantly greater improvements over time compared to the writing task in the following outcomes: a) relationship satisfaction, b) relationship confidence, c) individual well-being, d) hopefulness about the issue, e) confidence in resolving the issue, f) communication about the issue, and g) distress related to the issue. This between-group hypothesis is based on the expectation that participants will engage more fully with Amanda due to the interactive and responsive nature of the chatbot, potentially enhancing therapeutic outcomes. This may only show up two weeks later as participants will have had an opportunity to interact with their partner.

## Method

### Ethics statement

The study was approved by University of Lausanne’s Research Ethics Commission with the project number of C_SSP_052024_00015. Participants were provided with an informed consent form and were asked to tick an electronic checkbox to indicate their consent. They were told they could withdraw their participation at any time until the data collection was completed.

### Transparency and openness

The study was preregistered, and the preregistration can be found here: https://osf.io/wcghs . The analyses related to attitudes are reported in another manuscript [31]. All the study materials and analyses as well as the data can be found on the Open Science Framework project page: https://osf.io/wycda/. Session transcripts are not published openly due to the potentially sensitive and identifying information contained in the transcripts. We report how we determined our sample size, all data exclusions, all manipulations, and all measures in the study.

### Participants

**Fig 1:**
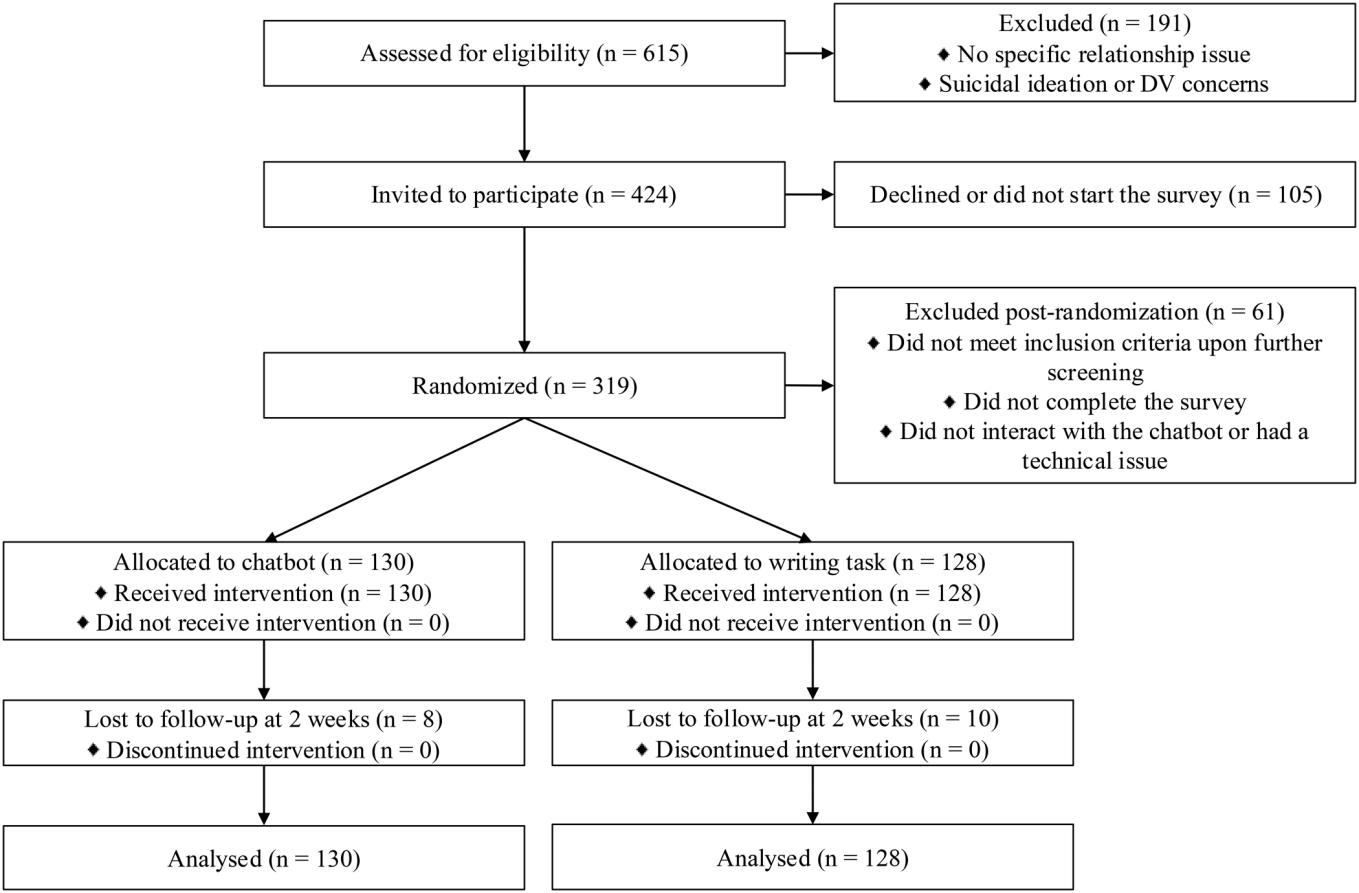
A Consort Diagram.

A total of 615 participants completed the initial eligibility survey. Of these participants, 191 were not eligible due to the potential risk level being too high (e.g., reported suicidal ideation or potential domestic violence) or them not reporting on any specific relationship issue that they wanted to address. The remaining 424 participants were invited to complete the study with 319 participants starting the survey. A further 61 participants were excluded because they either did not meet the inclusion criteria, did not complete the survey, did not interact or complete the interaction with the chatbot, or had an issue with the chatbot interaction (either they stopped the interaction or were not able to launch the chatbot). Thus, the final sample consisted of 258 participants (130 in the chatbot condition and 128 in the writing condition). Please see the consort diagram in Fig 1 for a visual representation. Most participants were women (n = 169, 81%), heterosexual (n = 221, 86%), married (n = 132, 51%), white (n = 205, 79%), lived in the UK (n = 227, 88%), and had at least an undergraduate degree (n = 197, 76%). Most participants had also had at least some experience with chatbots (n = 186, 72%) and/or virtual assistants (n = 225, 87%). See Table 1 for the details of demographic variables for each group. At follow-up we had a total of 240 participants (122 in the chatbot condition and 118 in the writing condition) resulting in a dropout rate of 7.7%.

**Table 1:** Demographic and Background Characteristics of Participants Across Chatbot and Writing Task Conditions.

	Chatbot (n = 130)		Writing Task (n = 128)	
Variable	*n*	*%*	*n*	*%*
Gender				
Woman	81	62.3	88	68.8
Man	48	36.9	40	31.3
Other	1	0.8	0	0.0
Having children				
Yes	62	47.7	68	53.1
No	68	52.3	60	46.9
Sexual orientation				
Heterosexual	110	84.6	111	86.7
Lesbian/gay	6	4.6	6	4.7
Bisexual	12	9.2	9	7.0
Other	2	1.5	2	1.6
Relationship status				
Dating	5	3.8	5	3.9
In a committed relationship	40	30.8	29	22.7
Cohabiting	21	16.2	26	20.3
Married	64	49.2	68	53.1
Highest level of completed education				
Middle school/secondary school	2	1.5	1	0.8
Some high school/secondary school	4	3.1	4	3.1
Graduated high school/A-levels	14	10.8	17	13.3
Some college/university	20	15.4	20	15.6
Undergraduate degree	64	49.2	51	39.8
Postgraduate degree (master’s and/or PhD)	26	20.0	33	25.8
Other	0	0.0	2	1.6
Current employment status				
Employed full-time	80	61.5	72	56.3
Employed part-time	20	15.4	17	13.3
Unemployed	8	6.2	10	7.8
Retired	2	1.5	2	1.6
Self-employed	8	6.2	12	9.4
Student	10	7.7	19	14.8
Other	2	1.5	6	4.7
Ethnicity				
White	109	83.8	96	75.0
Asian	5	3.8	13	10.2
Black	11	8.5	14	10.9
Mixed	4	3.1	4	3.1
Country of residence				
UK	118	90.8	109	85.2
Australia	4	3.1	7	5.5
Ireland	4	3.1	5	3.9
USA	3	2.3	7	5.5
Prior experience with chatbots (e.g., ChatGPT, Bard)				
Yes	92	70.8	94	73.4
No	38	29.2	34	26.6
Prior experience with virtual assistants (e.g., Alexa, Siri)				
Yes	114	87.7	111	86.7
No	16	12.3	17	13.3
	*M (SD)*		*M (SD)*	
Age	37.0 (11.2)		36.2 (10.2)	
Relationship duration in years	10.4 (8.2)		11.0 (9.4)	

*Note.* Not all percentages will add to 100% due to missing data.

### Procedure

Participants were recruited through Prolific, an online participant recruitment platform, in July 2024. Participants were eligible to take part in the study if they were at least 18 years old, currently in a romantic relationship, and reported a specific relationship issue they wished to address. Participants were excluded if they indicated recent thoughts of self-harm or suicidal ideation, as assessed by item 9 of the PHQ-9 [32], or if they reported any experiences of domestic violence, including physical harm or threats of harm from their partner [33]. Individuals were also excluded if they reported frequent emotional abuse, such as being regularly insulted, screamed at, or cursed at by their partner. Finally, participants who failed to report a specific relationship issue during the eligibility screening were excluded. These criteria were implemented to ensure participant safety and to confirm the appropriateness of a self-guided, low-intensity intervention. Participants who were deemed ineligible due to elevated risk were contacted via Prolific and provided with links to appropriate mental health support services. The participants who fulfilled our eligibility criteria were invited to participate in the main survey. Based on an *a priori* power calculation, 214 participants (107 per condition) were required to achieve 90% power to detect a small effect size (η² = 0.1) with p < .05. Based on our prior experience recruiting participants via Prolific for similar studies, we accounted for up to 20% attrition rate at follow-up to ensure sufficient power, we aimed to recruit a total of 256 participants (128 per condition).

In the main survey, the participants first completed questions about demographics, previous experience with chatbots or virtual assistants, attitudes toward chatbots and online interventions, and a range of clinical relationship and individual outcomes not reported in this manuscript. After the initial set of questionnaires, participants were randomly assigned into one of two conditions: interaction with a chatbot or a writing task of a brief social psychological intervention [30]. Participants were randomly assigned using Qualtrics’ built-in randomization function, which automatically allocated participants after they completed the baseline measures. This ensured that allocation was concealed from both participants and researchers during the enrolment and data collection process. Each intervention lasted between 15–30 minutes. The details of each intervention are described below. After the intervention, participants completed the same questions as before the intervention, except for demographics, as well as additional questions about the usability of the chatbot or the writing task depending on which condition they were assigned to. At the end, participants were provided with details of online counseling services in case they wanted to seek further support. After two weeks, participants were invited to participate in a follow-up survey where they again completed the same set of questionnaires and some additional questions about whether they had applied what they learnt from the interaction with their partner. data were collected between July 11^th^, 2024 and August 1^st^, 2024.

### Interventions

**Chatbot Interaction.** In the chatbot interaction, participants were asked to engage with Amanda, a chatbot created for the purpose of this study, about their specific relationship issue for 20–30 minutes until the conversation naturally came to an end. Amanda is based on OpenAI’s GPT-4o LLM and is programmed to have a “memory” of the conversation meaning that the conversation was appended at the end of the chatbot prompt so that it could follow the conversation. GPT-4o is similarly to GPT-4 not specifically trained to be a psychotherapist but Amanda received the following base prompt which allowed it to interact in a manner approaching a psychotherapist:

You are a trained psychotherapist called Amanda specialising in working with relationship difficulties. I would like you to respond as a relationship therapist: reflect what the client has said, provide validation and empathy, stay close to what the client says instead of overinterpreting them, and ask follow-up questions designed for you to better understand the situation. Do not provide answers that are too long, only ask one question at a time, and try to maintain a natural conversation like I would have with a therapist. The conversations should last at least 20 interactions and should eventually end up with some relevant suggestions for how to improve the issue, but this should only come towards the end of the conversation once the person has had enough time to explore their issue and you have a good understanding of the issue and feel you can offer personalised suggestions for help. You should not offer any advice until you have had at least 10 interactions with the client or patient. Avoid asking for any information that is identifying (e.g., do not ask addresses, names, or companies where they work). If they do not provide any context, assume you know nothing about their situation and ask them for more information.

**Writing Task.** To compare the chatbot intervention to another intervention, we selected the reappraisal writing intervention (Finkel et al., 2013) because it allowed us to match the chatbot condition as closely as possible in both duration and cognitive demand. Both interventions required participants to actively reflect on their relationship issue for approximately 20–30 minutes, supporting a meaningful comparison of efficacy and feasibility across conditions. The participants completed a set of four writing tasks each for at least 3 minutes and up to 5 minutes. In Step 1, participants were asked to write a fact-based summary of the most significant disagreement they had experienced with their spouse over the preceding 2 weeks for 5 minutes, “focusing on behavior, not on thoughts or feelings.” If they could not think of a conflict in the past two weeks, they were asked to describe a significant conflict that was as recent as possible.

In Step 2, Participants were asked to complete a writing task during which they reappraised the conflict they had just written about. During the reappraisal writing task, participants responded to three prompts (5 minutes for each prompt): (1) “Think about the specific disagreement that you just wrote about having with your partner. Think about this disagreement with your partner from the perspective of a neutral third party who wants the best for all involved; a person who sees things from a neutral point of view. How might this person think about the disagreement? How might he or she find the good that could come from it?” (2) “Some people find it helpful to take this third-party perspective during their interactions with their romantic partner. However, almost everybody finds it challenging to take this third-party perspective at all times. In your relationship with your partner, what obstacles do you face in trying to take this third-partner perspective, especially when you’re having a disagreement with your partner?” (3) “Despite the obstacles to taking a third-party perspective, people can be successful in doing so. Over the next 2 weeks, please try your best to take this third-party perspective during interactions with your partner, especially during disagreements. How might you be most successful in taking this perspective in your interactions with your partner over the next 2 weeks? How might taking this perspective help you make the best of disagreements in your relationship?”

### Measures

**Demographic information.** Participants completed a baseline questionnaire, which included questions on age, gender, ethnicity, sexual orientation, education level, employment, relationship status, children, country of residence, and relationship length.

**Chatbot experience.** Two single-item questions were used to assess participants’ previous experience with chatbots. These questions inquired if participants had used an AI-powered chatbot or virtual assistant (e.g., ChatGPT, Bing, Siri). Response options were “Yes” or “No,” with additional specifications if “Yes” was selected.

**Attitudes toward chatbots** [34]. A 14-item scale, divided into four subscales, measured participants’ attitudes toward chatbots: Performance Expectancy, Effort Expectancy, Willingness to Accept AI Devices, and Objection to AI Devices. Performance Expectancy included four items (e.g., “AI devices are more accurate than human beings”), while Effort Expectancy contained three items (e.g., “It takes me too long to learn how to interact with AI devices”). The Willingness and Objection subscales included three and four items respectively (e.g., “I am likely to interact with AI devices”; “I prefer human contact in service transactions”). Each item was rated on a 5-point scale, from strongly disagree [1] to strongly agree [5].

**Attitudes toward internet-based therapies** [35]. Participants’ views on internet-based therapies were assessed with 17 single-item questions, such as “Internet-based therapies are an appropriate alternative to conventional face-to-face psychotherapy.” Responses were rated on a 5-point Likert scale from strongly disagree [1] to strongly agree [5].

**Relationship satisfaction.** The Couple Satisfaction Index (Funk & Rogge, 2007), a four-item measure, assessed relationship satisfaction. Items included “In general, how satisfied are you with your relationship?” Overall scores ranged from 0 to 23, with higher scores indicating greater satisfaction.

**Relationship confidence.** Confidence in relationship trajectory was measured using a two-item scale adapted from [36]. Participants were asked “I believe we can handle whatever conflicts will arise in the future” and “I feel good about our prospects to make this relationship work.” Response options ranged from strongly disagree (scored as 1) to strongly agree [7], with higher scores reflecting greater confidence in the relationship.

**Dyadic coping.** The Dyadic Coping Inventory [37] assessed stress communication and coping strategies with partners. Two subscales were used: common dyadic coping (5 items, e.g., “We help one another to put the problem in perspective and see it in a new light”) and evaluation of dyadic coping (2 items, e.g., “I am satisfied with the support I receive from my partner and the way we deal with stress together.”). For the first subscale, higher scores indicate partners coping techniques when dealing with stress are better. For the second subscale, higher scores indicate a better personal evaluation of coping as a couple.

**Communication.** The Communication Patterns Questionnaire—Short Form (CPQ-SF; [38] was used to assess participants’ perceived frequency of communication patterns during conflict. The questionnaire consists of three subscales: constructive communication, self-demand/partner withdraw, and partner demand/self-withdraw. The constructive subscale consisted of three positive items and one negative item; a positive example item is “Both my partner and I try to discuss the problem”, and the negative item is “Both my partner and I blame, accuse, and criticize one another”. The self-demand/partner withdraw subscale contained 3 items; an example item is “I try to start a discussion while my partner tries to avoid a discussion”. The partner demand/self-withdraw subscale also contained three items; an example item is “My partner tries to start a discussion while I try to avoid a discussion”. Response options ranged from very unlikely (scored as 1) to very likely [9]. Each subscale was then calculated by adding or subtracting (for the negative item) the responses given for their respective items. Each subscale score ranges from 1 to 27, with a higher score corresponding to the perception of more frequent use of a specific communication pattern.

**Relationship issue assessment.** We asked participants five single-item questions regarding their current relationship issue. These questions assessed hopefulness (“How hopeful have you felt that you can resolve the relationship issue with your partner?”), confidence (“How confident have you felt that you and your partner will be able to overcome the relationship issue?”), and distress (“How distressed do you feel about the relationship issue?”). All items were rated from 0 to 6, with 0 being “not at all” and 6 being “extremely”.

**Relationship problem severity and confidence.** A single-item measure [39] evaluated the impact of the participants’ issue, rated on a 7-point Likert scale from 1 (not a problem) to 7 (extreme problem). Confidence in managing conflicts related to this issue was assessed similarly, with a 7-point scale from 1 (not confident) to 7 (extremely confident).

**Communication regarding relationship issues.** Two single-item questions asked participants to evaluate their communication and confidence in communicating with their partner about the relationship issue, both rated from 0 (not at all good) to 6 (perfect).

**Individual Well Being.** WHO-5 Well-being index [40] was used to measure participants’ current mental well-being. Participants rated how often they felt like items such as: “I have felt cheerful in good spirits.” Responses vary from “at no time” (0) to “all of the time” [5]. Overall scores range from 0 to 25, with higher scores indicating better mental well-being.

**The Chatbot Usability Questionnaire** (CUQ; [41] was used to ask participants with the chatbot condition to rate their experience with the chatbot. The questionnaire included 16 items, such as “The chatbot was easy to navigate” and “Chatbot responses were not relevant.” Responses were rated on a scale from 1 (strongly disagree) to 5 (strongly agree).

**Therapeutic skills.** Additionally, participants with the chatbot condition rated their experience using 17 single-item questions based off therapist ratings and qualitative interviews [27]. We conducted an exploratory factor analysis to assess the factor structure of the scale. The results showed that the scale items loaded onto one factor with item loadings above.3 except for Item 15 which was removed from the final scale. Therefore, the final scale consisted of 16 items. For participants in the writing condition, 10 single-item questions assessed the writing task’s effectiveness based on the same therapist ratings and qualitative interviews (e.g., “The writing task enabled me to explore the issue in depth”). The latter was not used in the manuscript as the purpose of the scale was only to make the two conditions more equal.

**Working alliance.** The Working Alliance Questionnaire [42] was used to evaluate the perceived alliance with the chatbot as a therapist. Participants rated six items, such as “The chatbot and I were working towards mutually agreed upon goals,” on a scale from 1 (not at all) to 6 (completely).

### Data analysis

Data were analyzed using a per-protocol approach, including only participants who completed the assigned intervention (i.e., interacted with the chatbot or completed the writing task) and provided post-intervention data. Participants who did not complete the intervention or encountered technical issues were excluded from analyses. The preregistered analyses were conducted using a 2 (group) x3 (time) mixed ANOVA for each outcome separately. When there was a significant interaction between group and time, we used Bonferroni corrected post-hoc tests to adjust for Type I error and understand at which timepoints the groups differed significantly. All analyses were conducted in *R*. The ANOVA analyses were conducted using *rstatix* package [43]. In our analyses, we used partial eta-squared (η²p) for the mixed ANOVA results, as it allowed us to isolate the effect sizes of individual factors while accounting for other variables in the model. For post-hoc tests, we reported eta squared (η²) as it provides a straightforward measure of the proportion of total variance explained by each group comparison. We interpreted effect sizes using the conventional cut-offs for eta-squared: small (0.01), medium (0.06), and large (0.14) [44]. For post-hoc tests, we applied the Bonferroni correction and used Cohen’s d as the measure of effect size using the conventional cut-offs: small (0.2), medium (0.5), and large (0.8) [44].

The technical outcomes of the session were analyzed using content analysis. A total of 21 codes were applied to the data. Eight codes were initially selected based on relevance from Abd-Alrazaq (2020) who conducted a review of the technical metrics to evaluate healthcare chatbots: dialogue efficiency, context awareness, error management, comprehensibility, realism, empathy, repetitiveness of response, and chatbot’s understanding of response. The scale was applied to all but one code, dialogue efficiency, in which the number of interactions was counted instead. 12 additional codes were developed by the first and third authors based on face validity and Vowels’ (2023) study to evaluate the therapeutic approach: repetitiveness of response, alliance, reflection, focus, therapeutic questioning, explorative approach, collaborative solutions, response length, overall sense of flow and coherence, addressing safety concerns, cultural adaptation, and privacy. The codes were rated on a scale from 0 (absent), 1 (sometimes present less than 50% of the time), 2 (mostly present more than 50% of the time) and 3 (completely present). The scale was applied to all codes except the last four, which were coded yes or no. The first and third authors individually applied these 21 codes to the data. Any discrepancies between coders were then discussed to 100% agreement, with most differences being between scores of 2 or 3.

## Results

### Clinical outcomes

The results for the clinical outcomes are available in Table 2. Across all outcomes, except for relationship confidence, the results consistently showed that both interventions (chatbot and writing task) improved problem-specific outcomes, generic relationship outcomes, and individual well-being over time. We conducted post-hoc tests to better understand when the improvements occurred over time. The results varied somewhat across the different outcome variables. The following variables improved from pre to post and remained stable at follow-up: hopefulness about the problem, confidence about the problem, problem conflict confidence, relationship satisfaction, and self-demand/partner-withdraw pattern. In other words, the results suggested that the participants improved from pre-intervention to post-intervention and this improvement stayed stable until two weeks later but did not further improve on these outcomes. There were also some outcomes that improved from pre- to post-intervention and continued to improve two weeks later: distress about the problem, perceived problem severity, communication about the problem, and constructive communication. There were also some variables which did not improve immediately after the intervention but improved two weeks later: common dyadic coping, evaluation of dyadic coping, partner-demand/self-withdraw pattern, and individual well-being. In summary, all outcomes except relationship confidence improved over time in both groups with the improvements always occurring from pre-intervention to two weeks later but for some outcomes also already improving already immediately after the intervention and for some outcomes continuing to improve between post-intervention and follow-up.

**Table 2:** Analysis of Variance (ANOVA) Results Comparing Chatbot and Writing Task Conditions Across Time Points for the Clinical Outcome Variables.

	Chatbot	Writing Task								
	M (SD)	M (SD)	Analysis (df)	F	*p*	η^2^p	a	Post-hoc (time)	*p.adj*	Cohen’s d
**Problem-specific outcomes**										
**Hopefulness about the problem**										
Pre	5.56 (1.48)	5.70 (1.21)	Group (1, 237)	0.55	.459	.002		Pre to post	**<.001**	.243
Post	5.84 (1.39)	5.89 (1.13)	Time (2, 474)	8.20	**<.001**	.033		Pre to follow-up	**<.001**	.197
Follow-up	5.66 (1.55)	5.94 (1.18)	Group * Time (2, 474)	1.84	.160	.008		Post to follow-up	1.00	.040
**Confidence about the problem**										
Pre	5.57 (1.54)	5.72 (1.26)	Group (1, 237)	0.49	.485	.020		Pre to post	**<.001**	.244
Post	5.86 (1.41)	5.89 (1.20)	Time (2, 474)	7.82	**<.001**	.032		Pre to follow-up	**.016**	.182
Follow-up	5.68 (1.57)	5.92 (1.27)	Group * Time (2, 474)	0.79	.453	.003		Post to follow-up	1.00	.056
**Distress about the problem**										
Pre	3.92 (1.62)	3.50 (1.65)	Group (1, 237)	3.90	**.049**	.016		Pre to post	**.017**	.173
Post	3.57 (1.77)	3.37 (1.62)	Time (2, 474)	24.92	**<.001**	.095		Pre to follow-up	**<.001**	.431
Follow-up	3.20 (1.65)	2.82 (1.51)	Group * Time (2, 474)	0.91	.403	.004		Post to follow-up	**<.001**	.281
**Problem severity**										
Pre	4.29 (1.64)	3.97 (1.50)	Group (1, 237)	2.45	.119	.010		Pre to post	**<.001**	.342
Post	3.84 (1.69)	3.61 (1.55)	Time (2, 474)	28.41	**<.001**	.107		Pre to follow-up	**<.001**	.454
Follow-up	3.56 (1.69)	3.40 (1.41)	Group * Time (2, 474)	0.77	.466	.003		Post to follow-up	**.012**	.188
**Problem conflict confidence**										
Pre	5.20 (1.44)	5.23 (1.37)	Group (1, 237)	0.32	.572	.001		Pre to post	**<.001**	.310
Post	5.70 (1.32)	5.48 (1.27)	Time (2, 474)	17.54	**<.001**	.069		Pre to follow-up	**<.001**	.364
Follow-up	5.64 (1.31)	5.62 (1.31)	Group * Time (2, 474)	1.26	.285	.005		Post to follow-up	1.00	.050
**Communication about the problem**										
Pre	4.63 (1.41)	4.62 (1.50)	Group (1, 237)	0.00	.967	.000	.75^a^	Pre to post	**<.001**	.453
Post	5.09 (1.46)	5.09 (1.33)	Time (2, 474)	47.30	**<.001**	.166	.85^a^	Pre to follow-up	**<.001**	.552
Follow-up	5.30 (1.39)	5.33 (1.27)	Group * Time (2, 474)	0.11	.892	.000	.90^a^	Post to follow-up	**<.005**	.205
**Generic relationship outcomes**										
**CSI - Relationship satisfaction**										
Pre	15.89 (4.62)	16.58 (4.25)	Group (1, 237)	0.83	.364	.003	.93	Pre to post	**.002**	.219
Post	16.30 (4.86)	16.75 (4.29)	Time (2, 474)	5.24	**.006**	.022	.96	Pre to follow-up	**.023**	.174
Follow-up	16.46 (5.16)	16.95 (4.40)	Group * Time (2, 474)	1.60	.203	.007	.92	Post to follow-up	.702	.077
**Relationship confidence**										
Pre	5.89 (1.27)	5.99 (1.11)	Group (1, 237)	0.05	.825	.000	.87^a^			
Post	6.03 (1.19)	5.98 (1.13)	Time (2, 474)	0.80	.449	.003	.91^a^			
Follow-up	5.87 (1.43)	6.03 (1.03)	Group * Time (2, 474)	1.41	.246	.006	.88^a^			
**Common dyadic coping**										
Pre	3.64 (0.84)	3.80 (0.89)	Group (1, 237)	0.29	.591	.001	.86	Pre to post	1.00	.016
Post	3.68 (0.87)	3.74 (0.94)	Time (2, 474)	5.84	**.003**	.024	.88	Pre to follow-up	**.024**	.172
Follow-up	3.77 (0.93)	3.83 (0.90)	Group * Time (2, 474)	0.97	.380	.004	.89	Post to follow-up	**.022**	.174
**Evaluation of dyadic coping**										
Pre	3.93 (0.99)	3.86 (0.90)	Group (1, 237)	0.64	.423	.003	.87^a^	Pre to post	1.00	.018
Post	3.94 (0.98)	3.84 (0.98)	Time (2, 474)	4.26	**.015**	.018	.94^a^	Pre to follow-up	**.047**	.157
Follow-up	3.96 (1.05)	3.99 (0.91)	Group * Time (2, 474)	1.40	.248	.006	.90^a^	Post to follow-up	.121	.133
**CPQ - Constructive communication**										
Pre	6.25 (1.50)	6.48 (1.57)	Group (1, 237)	0.02	.884	.000	.74	Pre to post	**.016**	.175
Post	6.54 (1.66)	6.50 (1.66)	Time (2, 474)	11.88	**<.001**	.048	.80	Pre to follow-up	**<.001**	.285
Follow-up	6.66 (1.68)	6.69 (1.50)	Group * Time (2, 474)	1.62	.199	.007	.82	Post to follow-up	**.044**	.159
**CPQ – Self-demand/partner-withdraw**										
Pre	3.61 (2.00)	3.83 (2.10)	Group (1, 237)	1.18	.278	.005	.79	Pre to post	**.010**	.185
Post	3.37 (2.12)	3.71 (1.98)	Time (2, 474)	7.52	**<.001**	.031	.83	Pre to follow-up	**.002**	.222
Follow-up	3.32 (1.93)	3.50 (1.80)	Group * Time (2, 474)	1.33	.265	.006	.75	Post to follow-up	.339	.103
**CPQ – Partner-demand/self-withdraw**										
Pre	3.11 (1.78)	3.16 (1.51)	Group (1, 237)	1.28	.259	.005	.68	Pre to post	.124	.128
Post	2.80 (1.69)	3.24 (1.74)	Time (2, 474)	4.15	**.016**	.017	.78	Pre to follow-up	**.034**	.165
Follow-up	2.75 (1.74)	3.05 (1.55)	Group * Time (2, 474)	3.65	**.027**	.015	.78	Post to follow-up	.651	.080
**WHO - Individual well-being**										
Pre	4.08 (0.96)	4.14 (1.03)	Group (1, 237)	0.11	.742	.000	.88	Pre to post	.780	.070
Post	4.11 (1.05)	4.15 (1.11)	Time (2, 474)	16.17	**<.001**	.064	.91	Pre to follow-up	**<.001**	.296
Follow-up	4.30 (0.98)	4.32 (1.08)	Group * Time (2, 474)	0.11	.899	.000	.92	Post to follow-up	**.004**	.253

*Note.* Bolded numbers indicate the analysis is statistically significant. a = Shows a Pearson’s correlation as only two items. Values in bold indicate significant results.

The results did not show any significant differences across groups apart from on two outcomes: The results showed that the participants in the writing task reported significantly lower distress about the relationship issue overall, including at pre-intervention. There was also a significant interaction between group and time on the CPQ – partner-demand/self-withdraw subscale showing that participants in the chatbot condition reported significantly lower levels of partner-demand/self-withdraw pattern immediately after the interaction with the chatbot (*p* < .046), but this difference was no longer significant at follow-up (*p* = .199). Thus, overall the results suggest that both interventions (chatbot and writing task) provide improvements in relationship and individual functioning.

### Feasibility outcomes

Table 3 reports the feasibility outcomes directly following the chatbot intervention. Overall, participants rated the chatbot highly on usability (4.19/5) and on therapeutic skills (3.99/5). They also rated their working alliance with the chatbot highly (4.75/6). Thus, the results suggest that the chatbot intervention provides a feasible option for offering relationship support, at least as a single session intervention. The main items that the participants rated lower included chatbot not clearly describing its purpose, it being somewhat robotic and moderately human-like, the responses were somewhat generic and repetitive, and the responses provided insights or new perspectives and explored the issue in depth only moderately. The rest of the attributes were rated overall very highly (above 4/5 or reverse scored items around 1/5).

**Table 3:** Post-Test Feasibility Outcomes for Chatbot Condition.

Questionnaire/item	Mean	SD	Min	Max
**Chatbot Usability Questionnaire (a = .87)**	**4.19**	**0.5**	**2.31**	**5**
1. The chatbot’s personality was realistic and engaging.	3.81	1.02	1	5
2. The chatbot seemed too robotic.	2.63	1.24	1	5
3. The chatbot was welcoming during initial setup.	4.32	0.68	2	5
4. The chatbot seemed very unfriendly.	1.33	0.56	1	4
5. The chatbot explained its scope and purpose well.	3.50	1.07	1	5
6. The chatbot gave no indication as to its purpose.	2.43	1.25	1	5
7. The chatbot was easy to navigate.	4.63	0.66	1	5
8. It would be easy to get confused when using the chatbot.	1.64	0.92	1	5
9. The chatbot understood me well.	4.10	0.94	1	5
10. The chatbot failed to recognise a lot of my inputs.	1.38	0.70	1	5
11. Chatbot responses were useful, appropriate and informative.	4.17	0.79	1	5
12. Chatbot responses were not relevant.	1.48	0.70	1	4
13. The chatbot coped well with any errors or mistakes.	3.98	0.90	1	5
14. The chatbot seemed unable to handle any errors.	1.58	0.91	1	5
15. The chatbot was very easy to use.	4.66	0.52	3	5
16. The chatbot was very complex.	1.63	0.93	1	5
**Therapeutic Skills (a = .93)**	**3.99**	**0.64**	**1.62**	**5**
1. The chatbot responses were helpful to address the issue.	4.35	0.73	2	5
2. The chatbot’s tone was very empathic.	4.33	0.79	1	5
3. The chatbot’s tone was very supportive.	4.46	0.66	2	5
4. I did not feel judged by the chatbot.	4.59	0.70	1	5
5. I felt listened to by the chatbot.	4.30	0.94	1	5
6. The chatbot responses felt too generic.	2.78	1.26	1	5
7. The chatbot thoroughly explored the issue.	3.88	0.92	1	5
8. The chatbot responses were repetitive.	3.15	1.32	1	5
9. The chatbot made me feel comfortable.	4.12	0.83	1	5
10. The solutions suggested by the chatbot were suitable for the issue.	4.31	0.74	1	5
11. The chatbot responses were relevant.	4.41	0.66	2	5
12. The chatbot responses were too long.	1.80	0.92	1	5
13. The chatbot provided me with new insights or perspectives on the issue.	3.64	1.01	1	5
14. The chatbot responses were human-like.	3.55	1.06	1	5
15. The chatbot took too long to respond.	1.20	0.49	1	4
16. The chatbot explored the issue in depth.	3.67	1.01	1	5
17. The chatbot enabled me to explore the issue in depth.	3.92	0.86	1	5
**Working Alliance Inventory (a = .93)**	**4.75**	**1.04**	**1.5**	**6**

### Technical outcomes

We coded all interaction transcripts between the participants and the chatbot for technical outcomes. We double coded 1/3 of the transcripts to ascertain reliability of the codes. We report reliability as percentage agreement between the two coders for each code. The overall Cohen’s Kappa was.868 across the coding between the two coders. Finally, the results for technical outcomes can be found in Table 4. The table also provides the percentage agreement between the two coders where the second coder coded 1/3 of the transcripts. The results showed that overall, the chatbot interactions were rated highly by the coders consistent with the ratings from the users. The main ratings where Amanda scored lower included it being repetitive and including herself in the process. All other items were overall rated at least 2.50/3. Finally, the chatbot explored a potential safety issue once out of three occasions where the coder(s) identified a potential safety concern that should be explored. When a participant described feelings of severe anxiety and low self-esteem or when another participant said they “feel like just giving up”, the chatbot did not address potential safety issues. However, when another participant talked about her sadness and crying a lot, Amanda explored coping mechanisms and suggested that the participant seek therapy for the feelings. When relevant, Amanda consistently addressed or accommodated participants’ culture in her responses. Finally, she never asked any identifying information from the participants and there was subsequently no identifying information in the transcripts.

**Table 4:** Technical Outcomes of Interaction Transcripts Coded by Researchers.

Code	Mean	SD	Median	Min	Max	Range	Rater % agreement
# of interactions	26.95	11.48	25	7	83	76	NA
Error management	2.98	0.12	3	2	3	1	95
Response length	2.98	0.12	3	2	3	1	92.5
Repetitiveness	2.08	0.67	2	1	3	2	75
Context awareness and memory	2.94	0.27	3	1	3	2	92.4
Comprehensible	2.95	0.26	3	1	3	2	95
Understanding the response	2.91	0.32	3	1	3	2	85
Realism/human-likeness	2.50	0.67	3	0	3	3	67.5
Including self	1.83	0.84	2	0	3	3	62.2
Empathy	3.00	0.00	3	3	3	0	97.5
Explorative approach	2.96	0.23	3	1	3	2	90
Collaborative	2.54	0.67	3	1	3	2	61.2
Focus	2.88	0.35	3	1	3	2	85
Reflection	2.97	0.21	3	1	3	2	100
Therapeutic questioning	3.00	0.00	3	3	3	0	100
Solution	2.66	0.59	3	0	3	3	78.9
Sense of flow and coherence	2.85	0.44	3	1	3	2	72.5
	Yes	No	NA				
Cultural adaptation*	4	0	126				
Addressing safety concerns*	1	2	127				
Privacy*	130	0					

*Note.* See descriptions of each of the codes in the online supplemental table S1 on the OSF Project page: https://osf.io/wycda

* Coded as yes/no. Cultural adaptation was only relevant if there was something in the transcript that may have required adaptation/addressing cultural issues (e.g., sexual orientation, religious practices. Safety concerns were only relevant in situations where the participant mentioned something the chatbot should have enquired about (e.g., user mentions major anxiety).

## Discussion

The results of this study were notably consistent, with 13 out of 14 outcomes showing significant improvements in relationship functioning, problem-specific measures, and individual well-being across both conditions, supporting H1. This indicates that both the chatbot and the writing task were effective in addressing key relational and personal outcomes. However, H2 was not supported, as no significant differences emerged between the two conditions for most clinical outcomes. These findings align with previous research suggesting that self-help interventions, including digital formats, can be just as effective as alternative options [10,11]. A possible explanation for the similar outcomes between the groups is that both interventions fostered emotional and cognitive reflection, which may be equally impactful in short-term, single-session formats. Although one of the anticipated advantages of chatbots is the potential to develop a therapeutic alliance that could enhance engagement [18,21], this study’s single-session design did not allow for the exploration of this dynamic. Therefore, further research is necessary to determine whether chatbots like Amanda can foster sustained engagement and long-term effectiveness in multi-session interventions.

Feasibility and technical outcomes have historically been underexplored in studies evaluating chatbot interventions, with only 1% of chatbot studies reporting technical outcomes and 37% reporting feasibility outcomes (Jabir et al., 2022). In the present study, participants rated the chatbot highly for usability and therapeutic skills, supporting the feasibility of using a GPT-4o-based chatbot like Amanda for relationship support. Specifically, users rated the chatbot highly in terms of empathy and comprehensibility, though some reported that the responses were occasionally repetitive or somewhat robotic (i.e., the responses followed a specific formula of reflection, validation, question). Furthermore, participants also rated therapeutic alliance with the chatbot highly, mirroring previous studies which found that users can develop a therapeutic alliance with chatbots which mirrors that of human therapists [16,22]. The coding of technical outcomes by researchers corroborated these user ratings, with high levels of agreement between the users and researchers. These findings are consistent with previous research [29], which highlighted the potential for chatbots to provide feasible relationship support, but also pointed out similar limitations, such as occasional lack of human-like nuance. While the chatbot performed well in technical aspects like error management and context awareness, the single-session design limited our ability to assess its long-term capabilities.

### Strengths and limitations

This study presents several strengths that contribute to the growing body of research on digital interventions for relationship support. One of the key strengths is its use of a randomized controlled trial (RCT) design, which ensures a high level of internal validity by minimizing selection bias and providing a robust comparison between the chatbot intervention and the writing task. Additionally, we also included a follow-up two weeks later which gave the participants time to apply the solutions they had decided on with the chatbot. Another notable strength is the comprehensive evaluation of clinical, feasibility, and technical outcomes, which allowed for a holistic assessment of the chatbot’s efficacy, user experience, and overall performance. Additionally, the use of a GPT-4o-based chatbot, Amanda, represents an innovative application of state-of-the-art AI technology, further advancing research on digital therapeutic tools.

However, this study also has several limitations that should be acknowledged. First, the single-session format limits the potential to assess one of the key advantages of chatbots over self-guided online interventions: their ability to build a therapeutic alliance over time. While chatbots like Amanda hold the promise of reducing dropout rates by fostering this alliance, we were unable to evaluate this potential in the current design. Another limitation is the non-clinical nature of the sample. Participants in this study were well above the cutoff score for clinically significant distress (13.5) and reported high levels of relationship confidence (nearly 6/7), which likely reduced the room for improvement and, consequently, the effect sizes observed. Furthermore, participants were paid for their time and were not actively seeking relationship interventions, limiting the generalizability of these findings to clinical populations. The sample’s willingness to engage with online interventions might also introduce selection bias, reducing the applicability of these findings to broader populations. A further limitation is that we used a per-protocol approach rather than an intention-to-treat analysis. While this allowed us to assess intervention effects among participants who completed the intervention as intended, it may overestimate efficacy by excluding those who disengaged. As such, the results may reflect ideal conditions rather than real-world effectiveness. Future studies should consider using intention-to-treat analyses to better capture the potential impact of dropout and incomplete engagement. Finally, Amanda is based on the LLM GPT-4o and we did not compare other high-performing LLMs such as Anthropic’s Claude 3.5 Sonnet, Google’s Gemini 1.5, or Meta’s Llama 3.0 which may provide different performances over GPT-4o.

### Future directions

Future research should explore the use of GPT-4-based chatbots in multi-session interventions to assess their ability to build a sustained therapeutic alliance and improve long-term outcomes. Given the limitations of the single-session format in this study, future studies should examine whether extended interactions with a chatbot can enhance engagement and reduce dropout rates, a common issue in digital interventions. Additionally, future trials should focus on clinical samples, including participants experiencing clinically significant distress, to better understand the efficacy of chatbot interventions in populations that more closely resemble those seeking therapeutic support. This would provide insights into how well these tools perform in real-world, high-need contexts. Another avenue for research could involve comparing different large language models (LLMs), such as GPT-4, Claude, and Llama, to identify which models are best suited for relationship interventions and mental health support. Finally, while this study focused on relationship support, future research could expand the scope to explore the feasibility and efficacy of chatbot interventions across other domains, such as individual therapy or family counseling.

### Implications for theory, research, and practice

The findings of this study have several important implications for theory, practice, policy, and research. Theoretically, this study reinforces the growing body of literature that suggests digital interventions, such as chatbots, are accessible, disseminatable, and have the potential to be an alternative to traditional face-to-face interventions assuming effect sizes grow as the number and length of sessions increase. This aligns with emerging theories on digital mental health, which emphasize the scalability and accessibility of AI-driven interventions. From a practical perspective, GPT-4-based chatbots like Amanda offer a promising tool for delivering relationship support at scale, particularly for individuals who face barriers to accessing in-person therapy. However, a critical concern highlighted by this study is the chatbot’s ability to identify and respond to safety risks. While Amanda explored potential safety concerns in a situation where a participant reported crying because of her sadness, she did not do so in examples where one participant described severe anxiety, or another said they feel like “just giving up”. We had screened out all participants with potential safety concerns, so no participant was in danger. However, the chatbot was not told that the participants had no safety concerns so should still have checked in with the participant. This underscores the importance of refining chatbot algorithms to better handle safety concerns, especially when used in mental health interventions. Policymakers should consider the need for robust safety protocols when considering integrating AI tools into therapeutic settings to ensure that vulnerable individuals are protected. One possibility is to integrate an “AI supervisor” with the chatbot therapist which is specifically instructed to only focus on potential risks that can take over the conversation when it identifies risk.

## Conclusion

In conclusion, the present study provided initial evidence of efficacy and feasibility of using an LLM-based chatbot for relationship support and showed that the chatbot displayed effective therapeutic skills. In the future, improving the chatbot’s ability to detect risk should be a priority, particularly when working with clinical populations who may be at greater risk of harm. Additionally, while this study demonstrated the efficacy and feasibility of single-session interventions, further exploration of how chatbots can support ongoing therapy and address safety in multi-session formats is crucial. By advancing chatbot technology and integrating more sophisticated risk detection mechanisms, AI-based interventions may become a safe and scalable option for both therapeutic support and crisis management.

**Data transparency statement:** The hypotheses related to the attitudes toward the chatbot and digital health interventions were analyzed and reported in a preprint which is currently under review: [blinded for peer-review].
